# Hock and Moselle

**Published:** 1907-07-06

**Authors:** Robert Gray

**Affiliations:** 6 Moorgate Street, London, E.C.


### HOCK AND MOSELLE.

Sir,—My letter to you, as published in to-day's Hospital, read with your editorial comments, appears to convict me of not knowing that "Ch. Smith-Haut-Lafitte" is a Red Graves. Few people, I venture to say, reading my letter as I wrote it and you received it, would take that view. I would ask you, as a matter of courtesy and fairness, to acquit me in your next issue of this lack of elementary knowledge of my own business, which I am ready to believe has been imputed by you under some misapprehension, and which none of my many friends in the trade would credit me with.

I referred in my letter, by implication, to the wine which in your statistical table on page 288 follows "Ch. Margaux"; I could not be expected to know that you meant "Ch. Smith-Haut-Lafitte," or, naturally, should not have spoken of it as a Médoc growth. It would, I readily allow, have been better to have referred to it as a Bordeaux wine, on the assumption that by "Ch. Haute Lafite" you might mean "Ch. Smith-Haut-Lafitte."

Yours truly,

Robert Gray.

6 Moorgate Street, London, E.C., June 29.

